# Supplementary material for: Signatures of single quantum dots in graphene nanoribbons within the quantum Hall regime
Source: Nanoscale. 2016 May 6;8(22):11480–6. doi: 10.1039/c6nr00187d (PMC5315012; doi:10.1039/c6nr00187d)
Supplement: Supplementary file 1 [file NR-008-C6NR00187D-s001.pdf]

Electronic Supplementary Material (ESI) for Nanoscale.
This journal is © The Royal Society of Chemistry 2016

Supplementary Information

for

Signatures of single quantum dots in graphene nanoribbons within the quantum Hall regime

Endre Tóvári,[a] Péter Makk,[b] Peter Rickhaus,[b] Christian Schönenberger,[b] and Szabolcs Csonka[a*]

1 Device geometry

The nanoribbons were fabricated by depositing a graphene flake over a bottom gate structure, then etching longitudinal edges and a hole in the center of the flake using reactive ion etching, before suspension. Fig. S1 shows an SEM image of a final double-ribbon structure. Two bottom gates - situated below the suspended branches and contacts - are schematically drawn in yellow. Conductance measurements as a function of the two gate voltages revealed that one branch was often dead in such devices. All measurements were performed in junctions with only one conducting branch.

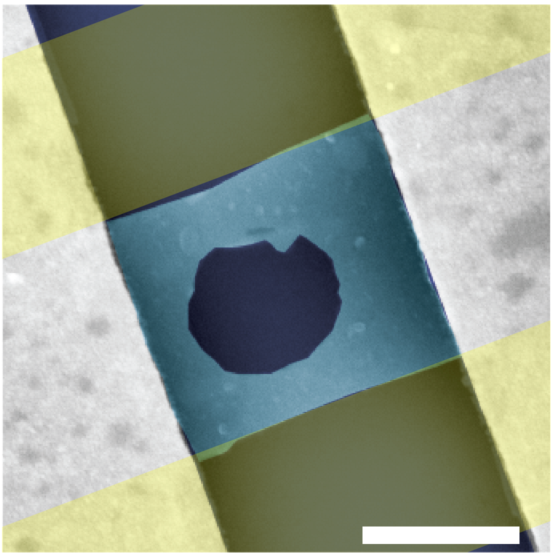

Figure S1: SEM image of a flake with a hole etched in the middle, from Ref. S 1. Bottom gates are drawn in yellow. Scale bar is 500 nm.

2 Coulomb diamonds on R1

$V_{SD} - V_g$ measurements at constant magnetic fields have also been performed on R1. Fig. S2 depicts Coulomb-diamonds at 4 T, belonging to conductance fluctuations near the gap edge.

[0a] Department of Physics, Budapest University of Technology and Economics, and Condensed Matter Research Group of the Hungarian Academy of Sciences, Budafoki út 8, 1111 Budapest, Hungary.
[0*] E-mail: csonka@mono.eik.bme.hu
[0b] Department of Physics, University of Basel, Klingelbergstrasse 82, CH-4056 Basel, Switzerland.

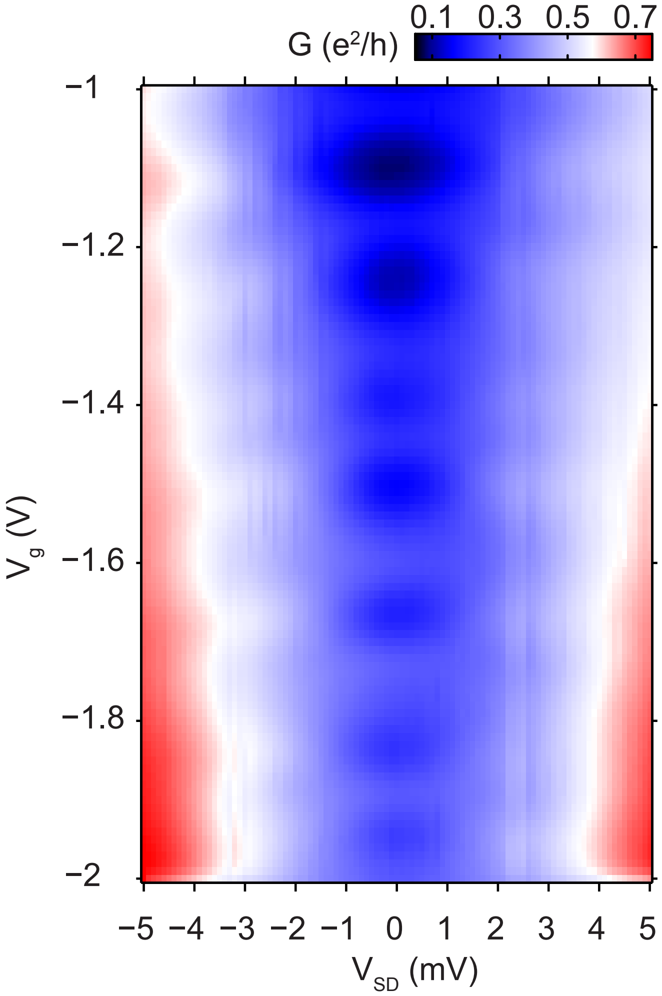

Figure S2: Conductance G of ribbon R1 at 4 T as a function of bias voltage V_{SD} and gate voltage V_g, exhibiting Coulomb-diamonds.

References

[1] R. Maurand, P. Rickhaus, P. Makk, S. Hess, E. Tovari, C. Handschin, M. Weiss and C. Schönenberger, Carbon, 2014, **79**, 486–492.
